# Evaluation of Peripapillary Nerve Fiber Layer after Dexamethasone Implantation (Ozurdex) in Branch Retinal Vein Occlusions

**DOI:** 10.1155/2016/2050796

**Published:** 2016-11-02

**Authors:** Muhammed Nurullah Bulut, Yusuf Özertürk, Ümit Çallı, Güzide Akçay, Ulviye Kıvrak, Kezban Bulut

**Affiliations:** Dr. Lütfi Kırdar Kartal Training and Research Hospital, Eye Department, Istanbul, Turkey

## Abstract

*Purpose.* To evaluate the peripapillary retinal nerve fiber layer (RNFL) thicknesses of patients treated with intravitreal Ozurdex implant due to branch retinal vein occlusion (BRVO) related macular edema (ME).* Methods.* Thirty-three eyes of 33 patients treated with Ozurdex implant due to ME associated with BRVO were included in the study. Ophthalmic examinations including determination of best corrected visual acuity (BCVA), measurement of intraocular pressure (IOP), and central macular thickness (CMT) and peripapillary RNFL assessment with optical coherence tomography (OCT) were performed before the injection of Ozurdex implant and during the 6-month follow-up period after the injection.* Results.* The mean age was 55.2 ± 7.4 (range: 40–68) years. The BCVAs were significantly increased and CMTs were significantly decreased at month 3 and month 6 visits compared to baseline values. The mean IOP was significantly increased from baseline at day 1, week 1, and month 1 visits (*p*_1_ = 0.008, *p*_2_ = 0.018, and *p*_3_ = 0.022, resp.). The average and inferior quadrant peripapillary RNFL thicknesses were significantly reduced at month 6 control visit compared to baseline values (both *p* < 0.001).* Conclusions.* Ozurdex implant improved the BCVA and reduced the CMT in the eyes with RVO related ME. However, IOP elevations occurred within the first month after the injection and the average and inferior quadrant RNFL thinning was found six months after the injection. Further controlled studies are warranted.

## 1. Introduction

Retinal vein occlusion (RVO) is the most common primary retinal vascular disorder which can involve the central retinal or branch retinal veins [1–3]. Macular edema (ME) is the main cause of visual impairment in both branch retinal vein occlusion (BRVO) and central retinal vein occlusion (CRVO) [4]. Several therapies including laser photocoagulation, antivascular endothelial growth factor (anti-VEGF) agents, and triamcinolone have been suggested for the treatment of ME associated with RVO [5].

Recently, Ozurdex (Allergan Inc., Irvine, CA, USA), a new sustained-release intravitreal dexamethasone implant, has been demonstrated to be effective in the treatment of patients with RVO related ME [6]. Ozurdex implant lowers the levels of many mediators that cause deterioration of blood-retina barrier, especially VEGF, and decreases the vascular permeability [7]. However, it has been shown to be associated with intraocular pressure (IOP) elevation [8–11].

Measurement of peripapillary retinal nerve fiber layer (RNFL) is used as a sensitive method for detecting early glaucomatous damage [12, 13]. In glaucoma, peripapillary RNFL is affected before the other glaucoma signs such as optic disc excavation and visual field defects occur [14]. Although Ozurdex implant has been found to cause IOP elevation [8–11], to the best of our knowledge, there is no study investigating the effect of this IOP elevation on peripapillary RNFL thickness. Therefore, in the present study, we aimed to evaluate the peripapillary RNFL thicknesses of patients treated with intravitreal Ozurdex implant due to RVO related ME.

## 2. Methods

Thirty-three eyes of 33 patients who were treated with Ozurdex implant due to ME associated with BRVO, in Kartal Education and Research Hospital Ophthalmology Department, between November 2013 and June 2014, were included in the study. Patients with ocular hypertension, glaucoma, neovascularization in angle, peripheral or macular ischemia on fundus fluorescein angiography (FFA), optic disc or peripapillary RNFL edema on optical coherence tomography (OCT) [15], and a history of antiglaucomatous drug use, intravitreal or subtenon corticosteroid injection, vitreoretinal surgery, or trauma were excluded. The study was in accordance with the rules of the Declaration of Helsinki and the approval of the Local Ethics Committee was obtained. All patients were informed about the study procedure and risks of the treatments and informed consents were obtained.

Each subject underwent a comprehensive ophthalmologic examination including determination of best corrected visual acuity (BCVA), measurement of IOP by applanation tonometry (same examiner), biomicroscopic evaluation, fundoscopy, gonioscopy, central macular thickness (CMT) and peripapillary RNFL assessment with OCT, and FFA before the injection of Ozurdex implant. BCVA was measured in Snellen decimals and converted to logarithm of the minimal angle of resolution (log MAR) for statistical analyses.

Ozurdex implant was injected to each eye intravitreally with its special applicator, from the pars plana region, in the operation room, under steril conditions. After the injection, topical antibiotics were given five times a day for one week. Follow-up visits were performed on day 1 and months 1, 2, 3, and 6. IOP was measured at day 1, week 1, and months 1, 3, and 6 visits. CMT and BCVA were evaluated at months 3 and 6 visits. Peripapillary RNFL was quantified at month 6 visit. The average and the superior, inferior, nasal, and temporal quadrant peripapillary RNFL thicknesses were measured by using spectral domain OCT. FFA was repeated at month 2 visit for all patients. Topical antiglaucomatous agents were started in patients who had IOP ≥ 22 mmHg at any control visit.

### 2.1. Statistical Analysis

Software SPSS version 22.00 (SPSS for Windows software; SPSS Inc., Chicago, IL) was used for statistical analyses. The distribution of variables was investigated by Kolmogorov-Smirnov test. Friedman test was used for analysis of the repeated measurements. Paired *t*-test was used for pairwise comparisons. Statistical significance was set at *p* < 0.05.

## 3. Results

The mean age of the study population was 55.2 ± 7.4 (range: 40–68) years. Eighteen patients were female and 15 patients were male. Thirteen eyes were pseudophakic and 20 eyes were phakic. The BCVA and CMT values of the patients are given in Table 1. The BCVAs were significantly increased and CMTs were significantly decreased at month 3 and month 6 visits compared to baseline values.

The IOP values are given in Table 2. The mean IOP was significantly increased from baseline at day 1, week 1, and month 1 visits (*p* = 0.008, *p* = 0.018, and *p* = 0.022, resp.). On the other hand, month 3 and month 6 IOP values were similar to baseline values.

Table 3 shows the baseline and month 6 peripapillary RNFL thicknesses of the patients. The average and inferior quadrant peripapillary RNFL thicknesses were significantly reduced at month 6 control visit compared to baseline values (both *p* < 0.001). There was no significant differences between month 6 and baseline superior, nasal, and temporal quadrant RNFL thicknesses. We did not observe any other side effect that could be related to the injections such as endophthalmitis, cataract development, or increase of previously available cataract.

## 4. Discussion

Our results displayed that although the BCVA significantly increased and the CMT significantly decreased in eyes with ME associated with BRVO during the 6-month follow-up period after intravitreal injection of Ozurdex implant, IOP elevations occurred within the first month after the injection and the average and inferior quadrant RNFL thicknesses were reduced at month 6 control visit.

Ozurdex implant is a biodegradable polymer including 700 *μ*g of dexamethasone and polylactic acid-polygcolic acid. It discharges dexamethasone in a biphasic manner. High concentrations occur within the first two months after the injection and then lower concentrations are seen after the third month [16]. In the current study, the IOP values were not higher than at baseline and at month 3 and month 6 control visits. However, it was significantly increased from baseline at day 1, week 1, and month 1 visits. This IOP elevation was in accordance with the pharmacokinetic characteristics of Ozurdex implant.

Previous studies have shown the steroid-induced IOP increases after the intravitreal injection of Ozurdex implant in the treatment of RVO related ME [8, 9, 11]. In addition, some studies have found an association between the secondary glaucoma and Ozurdex implant [10, 17]. However, in none of those previous studies [8–11, 17] peripapillary RNFL thicknesses of the patients have been evaluated. To our best notice, it is the first time in the literature we found the average and inferior quadrant RNFL thinning, despite use of topical antiglaucomatous agents in eyes with IOP ≥ 22, after the intravitreal injection of Ozurdex implant in eyes with BRVO related ME.

The pattern of RNFL thinning in glaucoma has the superior and inferior quadrant preference [18]. In our study, inferior quadrant RNFL was significantly thinner at month 6 visit than at baseline (*p* < 0.001). In addition, superior quadrant RNFL had also thinning; nevertheless, the difference could not reach the statistical significance (*p* = 0.059). Accordingly, it can be said that the month 6 peripapillary RNFL profile of the eyes was consistent with glaucomatous injury.

The RNFL thinning that was found in our patients could be due to the resolution of a BRVO related peripapillary RNFL edema, after the injection of Ozurdex implant. Since RVO causes blood flow blockage, a diffuse type of retinal edema may affect all retinal layers. Optic disc edema can be seen during the acute stage of RVO. Accordingly, swelling of the optic nerve head and the adjacent retinal tissue may affect OCT analyses [15]. All peripapillary RNFL values were inside normal limits according to the device's data base before the injections.

It has been reported that RVO related retinal ischemia may result in RNFL loss [19]. Lu and Zang [19] investigated the RNFL thicknesses of ischemic and nonischemic eyes with RVO and the fellow eyes. They found that retinal ischemia could cause RNFL defects, and the severity of those defects was correlated with the severity of ischemia. In our study, although we excluded the eyes with ischemia, we had not a control group to understand whether the RNFL thinning of our patients was due to the Ozurdex related IOP elevations or due to RVO related retinal ischemia. Lack of control group was the main limitation of the current study. The inclusion of a placebo group may be unethical in patients with RVO. However, a control group including RVO patients treated with anti-VEGF agents may be used in the future studies.

In conclusion, our results demonstrated that Ozurdex implant improved the BCVA and reduced the CMT in the eyes with ME associated with BRVO. However, IOP elevations occurred within the first month after the injection and the average and inferior quadrant RNFL thinning were found six months after the injection. On the other hand, it is not clear whether this RNFL thinning was caused by the Ozurdex related IOP elevations or BRVO related retinal ischemia itself. Further studies with appropriate control groups are promptly warranted.

## Figures and Tables

**Table 1:** The clinical characteristics of the patients (mean ± SD) (*n* = 42).

	Baseline	Month 3	Month 6	^*∗*^ *p* values
BCVA (log MAR)	0.9 ± 0.3 (range: 1.0–0.3)	0.4 ± 0.3 (range: 0.7–0.3)	0.3 ± 0.2 (range: 0.5–0.2)	<0.001
CMT (*μ*)	535.5 ± 93.8 (range: 315–778)	346.2 ± 58.4 (range: 266–508)	378.6 ± 57.6 (range: 266–522)	<0.001

BCVA: best corrected visual acuity; CMT: central macular thickness.

^*∗*^Friedman test.

**Table 2:** IOP changes before and after injection of Ozurdex.

	IOP	*p* value
Baseline	17.6 ± 1.7	
Day 1 after injection	18.7 ± 2.2	*p* = 0.008
Week 1 after injection	18.5 ± 2.2	*p* = 0.018
Month 1 after injection	18.4 ± 2.5	*p* = 0.022
Month 3 after injection	18.1 ± 1.6	*p* = 0.053
Month 6 after injection	18.1 ± 1.8	*p* = 0.053

*p* < 0.05: statistically significant.

**Table 3:** The peripapillary RNFL measurements (*μ*) of the patients (mean ± SD) (*n* = 42).

Quadrant	Baseline	Month 6	*p* value
Average	104.1 ± 11.1	101.1 ± 10.4	<0.001
Superior quadrant	121.7 ± 19.3	119.6 ± 19.3	0.096
Inferior quadrant	130.7 ± 17.5	126.4 ± 20.1	<0.001
Temporal quadrant	78.4 ± 12.9	77.2 ± 12.7	0.745
Nasal quadrant	85.3 ± 14.7	82.3 ± 11.8	0.459

RNFL: retinal nerve fiber layer.
